# Building on the COVID-19 in Pregnancy in Scotland study to support ongoing surveillance, research, and pandemic preparedness for maternal and child health

**DOI:** 10.7189/jogh.14.03046

**Published:** 2024-12-02

**Authors:** Rachael Wood, Sarah J Stock, Aziz Sheikh

**Affiliations:** 1 Public Health Scotland, Edinburgh, UK; 2 Usher Institute, University of Edinburgh, Edinburgh, UK; 3 Nuffield Department of Primary Care Health Sciences, University of Oxford, Oxford, UK

During the coronavirus disease 2019 (COVID-19) pandemic, Public Health Scotland and the University of Edinburgh led the COVID-19 in Pregnancy in Scotland (COPS) study [1]. COPS involved linking and analysing national vital events and health data sets held by Public Health Scotland to examine the impact of severe acute respiratory syndrome coronavirus two (SARS-CoV-2) infection and COVID-19 vaccination in pregnancy on maternal and baby outcomes from conception to the postpartum/neonatal periods. COPS was administratively linked to the Early Pandemic Evaluation and Enhanced Surveillance of COVID-19 (EAVE II) study [2] but had its own study data set.

COPS addressed key areas of policy and clinical uncertainty, with results quantifying the increased risk of specific outcomes following infection in pregnancy [3], demonstrating the low uptake of vaccination among pregnant women [4], and providing robust, population-based analyses showing the safety of vaccination in pregnancy [3,5,6] (**Table 1**).

**Table 1:** Key outputs from the COVID-19 in Pregnancy in Scotland study

Output	Key findings
Public Health Scotland official statistics [7]	COPS contributed data on infection and vaccination in pregnant women to Public Health Scotland’s official statistics on COVID-19 on a monthly basis, providing timely surveillance information to support the pandemic response in Scotland.
Early data on infection and vaccine uptake rates in pregnant women and associated short-term outcomes [4]	Low vaccine uptake rates in pregnant women compared to non-pregnant women in comparable age groups. A preliminary indication of increased risk of preterm birth and perinatal death within 28 days of maternal infection, but no increased risk after maternal vaccination.
Detailed analyses of pregnancy-related outcomes following infection and vaccination [3,5,6]	Suite of population-based, matched cohort studies showing association between infection and, separately, vaccination in pregnancy and: miscarriage and ectopic pregnancy; congenital anomalies; and perinatal outcomes for the mother and baby. No evidence of association between vaccination and any adverse outcomes. Evidence of an association between infection and specific adverse perinatal outcomes including maternal critical care admission, thrombo-embolism, and preterm birth.
Impact of infection with different viral variants on pregnancy-related outcomes [8]	Evidence of lower risk of adverse outcomes including maternal critical care admission and preterm birth following maternal infection in pregnancy when the Omicron, compared to the Delta, variant was dominant.
Confirmed infection in neonates [9]	Very low rate of confirmed infection in neonates over the first two years of the pandemic. Clinical outcomes following neonatal infection were good, with no neonatal deaths due to SARS-CoV-2 infection in Scotland over that time period.

COPS – COVID-19 in Pregnancy in Scotland study, COVID-19 – coronavirus disease 2019, SARS-CoV-2 – severe acute respiratory syndrome coronavirus 2

Several factors facilitated the delivery of COPS, including Scotland’s high-quality, population-based administrative data sets, expedited governance procedures facilitating their use for pandemic-related analyses, and established collaborations between Public Health Scotland and academics, which allowed the rapid assembly of the study team.

The key challenge faced by COPS was the lack of a preexisting, near-real-time, population-based e-cohort of all pregnancies, including ongoing pregnancies and all completed pregnancies regardless of duration or outcome, with records for mothers linked to those for the baby for pregnancies ending in a live birth. Such a cohort is a prerequisite for meaningful examination of associations between infection and vaccination and the full range of pregnancy-related outcomes, including early pregnancy, perinatal outcomes, and congenital anomalies.

At the start of the pandemic, records relating to pregnancies and births were contained in multiple disparate national data sets, reflecting different stages of pregnancy (ongoing or completed) and different pregnancy outcomes (e.g. miscarriage, termination of pregnancy, live or stillbirth), with some records relating to the mother and some to the baby. The first substantial phase of the COPS study, therefore, involved drawing on all relevant national vital event and health data sets to establish a comprehensive, intergenerational Scottish pregnancy and births e-cohort. Whilst ultimately underpinning all the COPS analyses, development of the cohort was time-consuming. Needing to create it from scratch introduced substantial delay before COPS could deliver substantive results.

COPS was a time-limited study with specific governance approvals related to the pandemic. Since it ended in September 2022, we have sought to consolidate the learning acquired through COPS to ensure that Scotland is optimally prepared to deliver timely surveillance and research on any future emerging threats affecting pregnant women. Specifically, Public Health Scotland has developed the Scottish Linked Pregnancy and Baby Data set (SLiPBD). Similar to the COPS cohort, SLiPBD is a population-based e-cohort of all pregnancies and births in Scotland based on linkage of existing national data sets. SLiPBD includes pregnancies from 2000 onwards. It was launched in September 2023 and is updated on a monthly basis [10,11].

It is not feasible to develop and maintain a new national data asset like SLiPBD on a ‘just in case' basis, not using it unless or until a new emerging infection or future pandemic occurs. SLiPBD has therefore been designed as a ‘foundation stone’ data asset which will underpin a range of ongoing business as usual analyses undertaken as part of Public Health Scotland’s core function, as well as enabling bespoke research analyses and any analyses required in response to emerging threats. The key purpose of SLiPBD is to enable rapid identification of pregnancy or birth (sub)cohorts required for specific analyses. In line with this, only minimal data items relevant to cohort identification are retained within SLiPBD, including unique patient identifiers, relevant dates, sociodemographic characteristics, and pregnancy outcome. As all the national vital event and health data sets held by Public Health Scotland also include unique patient identifiers, additional variables from these data sets can then be linked to SLiPBD records as required for specific analyses. Examples of existing national health data sets that can be linked to SLiPBD include prescribing, hospital discharge, and death records for mother and/or baby. In the event of a future pandemic, Public Health Scotland would be responsible for rapidly developing national data returns providing relevant novel data, as was done during the COVID-19 pandemic to provide information on viral testing and vaccination.

At present, Public Health Scotland is using SLiPBD linked to other national data sets for analyses relating to the uptake of vaccines routinely offered in pregnancy; prescription of potentially teratogenic medicines in pregnancy; coverage of and outcomes following pregnancy screening; and to enable surveillance of infections and environmental exposures in pregnancy. Extracts of SLiPBD, again linked to other national data sets as required, can be provided to external research teams subject to governance approvals through the NHS Scotland safe haven facility. Interested researchers should contact Research Data Scotland (see Data availability statement for details).

The creation and maintenance of a substantial new data asset like SLiPBD brings inevitable challenges. Whilst the need to learn from the COVID-19 pandemic has been widely acknowledged, in practice post-pandemic fatigue and funding constraints make this difficult. Ongoing, committed leadership is required to ensure that practical improvements are made and sustained. SLiPBD relies on the quality of the national vital event and health data sets that it draws on. The health service must continue to invest in the technology and the people, including administrative and clinical coding staff in local services, that contribute to these. The development of SLiPBD is not a one-off process: opportunities for continuous improvement should be sought. Specific opportunities that we hope to see over time include improved recording of ethnicity on source national data sets and hence within SLiPBD; new national data returns from early pregnancy clinics and improved access to primary care data to further enhance ascertainment of pregnancies ending in an early loss; and possibly a ‘research ready’ version of SLiPBD mapped to a common data model such as that provided by the Observational Medical Outcomes Partnership to facilitate federated analyses of pregnancy and birth cohorts from multiple settings.

Whilst the development and maintenance of SLiPBD has been the responsibility of Public Health Scotland, the COPS study was jointly led by Public Health Scotland and the University of Edinburgh, with academics from other universities making valuable contributions to the wider study team. The extent of the collaboration between Public Health Scotland as the national public health agency and the academic sector during the COVID-19 pandemic was a vital ingredient enabling rapid, robust analyses that critically informed policy and service decisions. Another question is therefore how such collaboration can be nurtured to ensure relationships and governance structures are in place to enable a rapid joint response to future threats. Joint appointments and honorary contracts are likely to be useful in this regard.

Overall, it is vital that we learn from the successes and challenges of the COVID-19 pandemic. By strategically improving our national data assets now, we will ensure that national public health agencies and academics are best placed to deliver timely policy and clinically relevant analyses when the next pandemic emerges.
